# Is population ageing cancelling out progress made in tuberculosis control in Hong Kong Special Administrative Region SAR (China)? Age-adjusted analysis of case notification data, 1990–2015

**DOI:** 10.5365/WPSAR.2016.7.3.011

**Published:** 2017-02-10

**Authors:** Jun Li, Nobuyuki Nishikiori, Chi Chiu Leung, Engkiong Yeoh, Puihong Chung

**Affiliations:** a JC School of Public Health and Primary Care, Chinese University of Hong Kong, Hong Kong SAR, China.; b World Health Organization Regional Office for the Western Pacific, Manila, Philippines.; c Tuberculosis and Chest Service, Department of Health, Hong Kong SAR, China.

## Introduction

For most countries and areas in the World Health Organization (WHO) Western Pacific Region, the decline of tuberculosis (TB) epidemics and the ageing of the population occurred simultaneously in the past decades. According to latest reports, people aged 60 years and over accounted for 13% of the population in 2010 in the Region, and the number will grow faster due to longer life expectancy and declining fertility. (*1*)

The impact of population ageing on TB epidemiology is complex and may vary between and within countries. In some high- and middle-income settings, like  Hong Kong Special Administrative Region SAR (China), the TB notification rate had declined slowly after a rapid downward trend. (*2*) Consistent high TB prevalence and incidence in older people is one potential reason and is increasingly becoming an important public health challenge. (*3*)

In Hong Kong Special Administrative Region SAR, one study demonstrated the TB rate decreased in those under 60, remained unchanged in those between 60 and 69 and increased in those more than 70 years of age from 1989 to 1998. (*4*) Tackling the challenge of an ageing population appears to be a key step for TB elimination. This report analyses surveillance data of TB notifications in Hong Kong Special Administrative Region SAR from 1990 to 2015 and discusses the impact of population ageing on achieving the WHO End TB Strategy targets. (*5*)

## Methods

TB has been a statutory notifiable disease in Hong Kong Special Administrative Region SAR since 1939. (*3*) Based on TB notification systems, the information of registered TB patients is collated and compiled in annual reports of the Tuberculosis and Chest Service, Department of Health, Hong Kong Special Administrative Region SAR. (*2*) We extracted the number of all forms of TB notifications by age and sex between 1990 and 2015. The number of the corresponding population was extracted from online publications of population estimates released by Census and Statistics Department, Hong Kong Special Administrative Region SAR. (*6*)

Descriptive analysis of TB rates from 1990 to 2015 was conducted. The age-specific TB rates by sex were analysed to compare the trends in each age group. In addition to crude TB rates, age-adjusted rates from 1991 to 2015 were calculated by using the population in 1990 as reference. The annual rate of reduction in TB notification was determined by fitting an exponential linear regression model for crude and age-adjusted TB rates respectively from 1998 to 2015. Then each fitted model was extrapolated up to the year 2035 to estimate and examine future TB rates in line with the End TB Strategy target (90% reduction in incidence by 2035 compared to 2015 level). All analyses were conducted by the statistical software environment R version 3.3.1 (R Core Team, Vienna, Austria, 2016).

## Results

The proportion of older people (people aged 65 years and over) in the population increased from 8.5% in 1990 to 15.3% in 2015, while the proportion of older TB patients increased from 21% in 1990 to around 40% in 2004 and subsequent years.

An overall downward trend of TB rates was observed in all age and sex groups after 2000 (**Fig. 1a**). The rates in older people were significantly higher than younger groups in both males and females. The rate in males was not obviously different from the rate in females in people under 35. However, the rate in males increased faster than that of females after age 35. In females, the rates between 15 and 34 years of age were conversely higher than those between 35 and 54 years of age.

**Fig. 1a:**
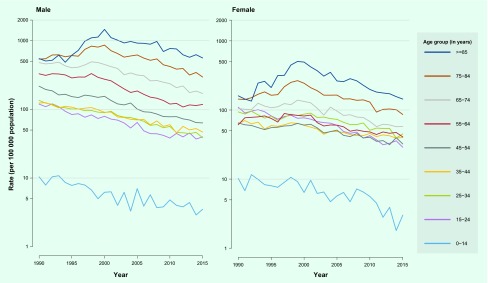
Tuberculosis notification rate by age and sex, Hong Kong Special Administrative Region SAR (China), 1990–2015^*#^

* Data in 2015 are provisional.

^#^ All forms of tuberculosis were included in the analysis.

The annual decline was an average of 3.9% per year in crude TB rates (3.7% in males and 3.9% in females); the decline was 5.4% per year in age-adjusted TB rates (5.7% in males and 4.9% in females) from 1998 to 2015 (**Fig. 1b**). Extrapolating this trend, the crude and age-adjusted rates were expected to reach 28.0 and 15.0 per 100 000 in 2035, which would result in a total reduction of 54.5% and 66.2% compared to the rates in 2015.

**Fig. 1b:**
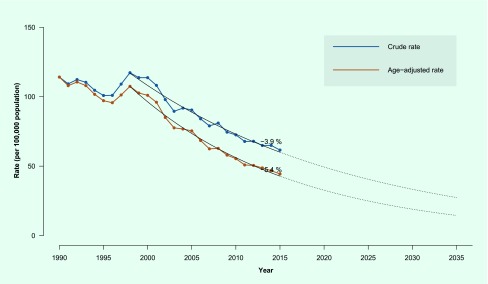
Crude and age-adjusted tuberculosis notification rates, Hong Kong Special Administrative Region SAR (China), 1990–2015^* #^

* Data in 2015 are provisional.

^#^ All forms of tuberculosis were included in the analysis.

## Discussion

The results demonstrate Hong Kong Special Administrative Region SAR age-specific TB rates in recent years. Along with implementation of the DOTS strategy, TB associated with progressive primary infection or exogenous reinfection had been well reduced in the community. (*3*) However, the diseases developing from endogenous reactivation were less affected. (*3*) Previous studies in Hong Kong Special Administrative Region SAR elaborated on the transition from high TB risk to far lower risk in young adults and TB rate increases with age in all birth cohorts after 1978. (*7*, *8*) Accordingly, the proportion of TB reactivation was estimated to increase from 46% to 70% between 1968 and 2008, (*9*) and almost to 100% by 2000 for the 65–74 years age group. (*10*) Older people are more likely to be infected in their earlier adult years and reactivate TB due to decreased immunocompetency. This may explain consistently higher rates in older people and the increasing TB trend with age in Hong Kong Special Administrative Region SAR.

The rates in males are obviously higher, probably due to more exposure and high-risk factors for progression such as comorbidity, smoking or alcohol abuse. (*11*) Higher rates among young to middle-aged women have also been observed in industrialized countries during the mid-twentieth century and in China in the past decades. Potential reasons, such as stress of pregnancy or immigration of female workers, warrant further studies. (*11*)

Overall, the impact of population ageing on TB rates seems substantial in Hong Kong Special Administrative Region SAR. When ageing progresses together with a decline in TB rates, the former would partially cancel out the progress by slowing down the reduction of TB rates as observed in Japan after the 1980s. (*12*) In Hong Kong Special Administrative Region SAR, the epidemiologic transition may take several decades in line with the demographic changes. Towards the End TB Strategy targets, although the decline of TB rates can be positively accredited, an additional 12% reduction would be lost exclusively ascribed to population ageing. In addition, the extrapolation should also consider the quality of current TB data, population estimated, declined annual risk of infection and a smaller proportion of infected migrants in subsequent birth cohorts. (*7*, *8*)

Therefore, a more targeted response is needed to move towards the End TB Strategy targets. Considering the limitation of existing tools for diagnosis and treatment, preventing reactivation from higher prevalence of latent TB infection in older people will remain a major challenge. Enhanced surveillance together with age-sensitive analysis particularly focusing on older people is critical to accurately monitor the situation under demographic changes, including migration, that are happening in Hong Kong Special Administrative Region SAR and other parts of Asia.
